# On Wounds of the Thorax and Abdomen

**Published:** 1818-06

**Authors:** 


### *On Wounds of the Thorax and Abdomen.* By Deputy Inspector HENNEN.

( Hennen's Military Surgery. )

### I. WOUNDS OF THE THORAX.

1. *Symptoms.* The same degree of obscurity which attends wounds of the head, and renders their pathology so difficult, does not equally obtain in those of the thorax. The injuries here are more cognizable to the senses, their symptoms more obvious, their treatment more determinate, and less complicated. Yet still the wounds of this important division of the human frame are as frequently fatal as those of the head. A ball, in striking the body or a limb, will often run round under the skin, and appear to penetrate right across the member or cavity. Thus Mr. H. has traced, by dissection, a ball passing into the thorax, making the circuit of the lungs, and emerging at the opposite side, giving the appearance of the man having been shot through the body, while bloody sputa seemed to prove the fact, and in reality rendered the same measures, to a certain extent, necessary.

Ball or bayonet wounds of the thoracic integuments require great attention from the danger of inflammation spreading to the pleura, or subjacent viscera, or of troublesome abscesses forming.

"When a ball (says this able surgeon) has fractured one or more ribs, we must not be contented by enforcing a strict diet, but we must call in the lancet to our aid, and keep the bowels freely open with mild purgatives, and the skin in a perspirable state by antimonials and diluents; aiding our endeavours with a supporting bandage, and picking away any spiculæ [spicula] of bone which are within our reach. In every injury of the chest, a firm elastic bandage is an indispensable assistant in the cure; the motions of the ribs are not only restrained, but the parts are powerfully supported by its application. If fracture has taken place in any of the bones, we have no other means so perfect of retaining them in their place; if a slight degree of emphysema has occurred before we see the patient, we thus prevent its further diffusion; and if we are called on before it takes place, *we may prevent the occurrence altogether.* The extent and tightness of this bandage should be such as to oblige the patient to perform respiration, as much as possible, by the aid of the diaphragm and abdominal muscles. If there be a wound, an opening ought to be left, so as to permit the usual dressings without removing the bandage." P. 394.

The indications of a wound penetrating the lung itself, are sufficiently well marked. *Immediate* bloody expectoration, dyspnœa, stricture and suffocation, insupportable anxiety and faintness, soon discover the fact. The great danger is the debility from excessive internal or external hæmorrhage, or of suffocation. The effusion of air forming emphysema is also a troublesome, but taking it abstractedly, not a dangerous symptom. The consecutive symptoms are, violent inflammatory affections of the lungs and membranes; long and tedious suppurations and exfoliations of the bones; cough, emaciation, and hectic fever.

2. *Treatment.* In whatever part of the thorax a ball, bayonet, or sabre may strike, our first object is to diminish the volume of the circulation. On this, the very existence of our patient depends.

" There lies a man with a wound of his chest; the blood is oozing from the external orifice in a constant, though slow florid track; in his frequent and painful efforts to cough, it is thrown up in frothy arterial mouthfuls, mixed with occasional clots; his breathing is oppressed almost to suffocation; his pulse weak, quick, and fluttering; his eyes are starting from their sockets; his nostrils are distended in his efforts at relief by inspiration; his extremities are cold, and often tossed about in fruitless anguish. This wretched being must assuredly die, if surgical aid be not promptly afforded him." P. 397.

Without searching after balls or fragments of bones, or discussing, as young men fresh from their studies are wont to do, respecting the vessels which may probably be wounded, let the patient be laid down in a cool place, and thirty or forty ounces of blood immediately abstracted from the arm by a large orifice. Should he faint under this process, instead of administering cordials, let the finger be introduced into the wound, and any extraneous matter extracted. If the orifice be narrow, dilate it cautiously. If a gun-shot wound, dress mildly and superficially; if an incised one, let the lips be closed at once, which will be the surest preventive of emphysema, hæmorrhage, or collections of matter. After an interval of quiet, the vessels will again begin to pour forth their contents, with spitting of blood, &c.; and again the lancet must be used. If we get our patient through the first twelve hours, we have reason to hope that he will not die of hæmorrhage; nothing but venæsection is to be employed against this source of danger. We may again repeat, that the closure of the wound is an important point of surgical practice in these cases.

When the first rush of hæmorrhage has abated, we may have recourse to substitutes for the lancet. Digitalis, and, when the cough is troublesome, opiates, with rigorous diet, amounting to the total prohibition of every thing solid, and admitting but mild and unirritating liquids. Gentle laxatives, perfect tranquillity, and cool air are essential requisites here.

### II. Wounds of the Abdomen.

Most of the directions given above are equally applicable here. In penetrating wounds of the abdomen, if no protrusion takes place, the lancet, with its powerful auxiliaries—abstinence, rest, supine posture, are our chief dependence. Great pain and tension must be relieved by leeches, fomentations, warm bath, and the mildest aperients, particularly oily glysters. It is astonishing what Nature will sometimes effect in injuries of the intestines!

"Among others (says Mr. Hennen), I have been a witness to the recovery of a soldier who had been shot through the abdomen by a ramrod, which passed in anteriorly, and actually stuck in one of the transverse processes of the vertebræ, from which it was not disengaged without some force."

This man recovered.

"The apprehensions of abdominal effusions are now pretty well subdued. The occurrence is extremely rare; and when it does happen, we leave the poor wretch to die in peace, without searching after effused fluids, the nature of which cannot be known; or, if known, the information cannot, in the most remote degree, lead to recovery." P. 448.

In respect to the application of sutures in wounded intestines, Mr. H. "having only practised the mode by a single stitch to the abdominal parietes, and then closing the wound," he can only speak of that alone.

*Conclusion.* "It will be obvious (says Mr. H.) in the perusal of these sheets, how much I have trusted to DEPLETION; but I beg to remind my readers, that I have been describing the injuries of robust young soldiers, full of life and vigour, and fitted for all the purposes of active warfare; living principally in the field; enjoying few, if any, of the luxuries of domestic society: and, consequently, exempted from many of the diseases incident to the inhabitants of cities. A short absence from the army has, however, been often attended with a remarkable change in their constitution; the men who were once in the hospitals in the rear, have almost constantly formed the great majority of their inhabitants afterwards. It may be said, they were weakened by previous sickness. To a certain extent, I admit the fact; but the same cannot be said of the officers who remained in the Depôts, from duty or other causes, and who preserved their health there. These men could not bear the privations of the field; they were subject to low typhoid febrile attacks; they could not bear evacuations, either in their diseases or their wounds, to any thing like the extent of those more actively employed. The difference of success was so notorious, that the Depôt officers were consigned to certain death when they joined the army, by their veteran brethren; but what military men deemed judgments, medical men accounted for upon physical principles." P. 490.

As penetrating wounds of the thorax and abdomen are not very rare occurrences in private practice, we have thought it useful to condense a few passages from so recent and respectable a work as Mr. Hennen's, on these interesting points, especially as we passed them over in our former review of the book, which we again recommend to the attention of the surgical reader.

---

